# Bacteria–phage (co)evolution is constrained in a synthetic community across multiple bacteria–phage pairs

**DOI:** 10.1099/mic.0.001577

**Published:** 2025-06-19

**Authors:** Meaghan Castledine, Daniel Padfield, Marli Schoeman, Amy Berry, Angus Buckling

**Affiliations:** 1 College of Life and Environmental Sciences, Environment and Sustainability Institute, University of Exeter, Penryn, Cornwall, TR10 9EZ, UK; 2 Falmouth School, Trescobeas Rd, Falmouth TR11 4LH, UK

**Keywords:** bacteria–phage (co)evolution, bacteriophage, coevolution, community ecology, evolutionary ecology, phage resistance

## Abstract

Bacteriophages can be important drivers of bacterial densities and, therefore, microbial community composition and function. These ecological interactions are likely to be greatly affected by evolutionary dynamics because bacteria can rapidly evolve resistance to phage, while phage can reciprocally evolve to increase infectivity. Most studies to date have explored eco-evolutionary dynamics using isolated pairs of bacteria–phage, but in nature, multiple bacteria and phages coexist and (co)evolve simultaneously. How coevolution plays out in this context is poorly understood. Here, we examine how three coexisting soil bacteria (*Ochrobactrum* sp., *Pseudomonas* sp. and *Variovorax* sp.) interact and evolve with three species-specific bacteriophages over 8 weeks of experimental evolution, both as host–parasite pairs in isolation and as a mixed community. Across all species, phage resistance evolution was inhibited in polyculture, with the most pronounced effect on *Ochrobactrum*. Between bacteria–phage pairs, there were also substantial differences in the effect of phage on host densities and evolutionary dynamics, including whether pairs coevolved. Our results also indicate bacteria have a relative advantage over phage, with high rates of phage extinction and/or lower densities in polyculture. These contrasts emphasize the difficulty in generalizing findings from monoculture to polyculture and between model bacteria–phage pairs to wider systems. Future studies should consider how multiple bacteria and phage pairs interact simultaneously to better understand how coevolutionary dynamics happen in natural communities.

## Data Availability

R code and data are deposited on GitHub (github.com/mcastledine96/Polyculture_suppresses_coevolution_2024).

## Introduction

Bacteriophages (‘phages’), as the most abundant ‘life form’ on Earth, are assumed to come into frequent contact across natural environments. To defend themselves from infection, bacteria can evolve to become phage-resistant, while phages can also evolve to overcome resistance and regain infectivity (coevolution) [1,3]. Coevolution can have profound impacts on bacteria–phage evolution and ecology, including driving population dynamics [4,5] and the evolution of virulence of bacterial pathogens [6,8]. While there is mixed evidence of coevolution in natural populations [9], bacteria–phage coevolution has been unequivocally demonstrated in several model systems, including *Escherichia coli* [10], *Pseudomonas* (*Pseudomonas fluorescens* [11] and *Pseudomonas syringae* [12]) and *Staphylococcus aureus* [13]. However, how ubiquitous rapid coevolution is across non-model systems and in more complex contexts than monoculture (single bacteria and phage pairs) is poorly understood.

(Co)evolutionary dynamics can be greatly affected by environmental conditions including the presence of competitors [14,15]. A general expectation is that pairwise reciprocal selection will be weakened in more complex communities. As densities are typically lower in communities, this will reduce encounter rates and, therefore, selection for resistance and infectivity [16,17]. Reduced densities and slower growth rates will reduce mutation supply rates, thereby further slowing (co)evolution [1,14, 18,20]. Furthermore, competitors and parasites/predators can result in trade-offs in resistance to phage and maintaining competitive ability and/or defences to predators [21,23].

While studies have characterized bacteria–phage (co)evolution with conspecifics (including in soil [14], plant leaves [24], digestive tracts [25,26] and marine systems [27]), these studies typically focus on a single bacteria and phage pair [14,22, 26, 28, 29], whereas in nature multiple bacteria–phage pairs coexist together. How bacteria evolve resistance (and phages reciprocally evolve infectivity) may not be equal among coexisting community members and their respective phages. Bacteria populations that are more dense or faster growing are likely to experience greater encounter rates and, therefore, be under more selection for resistance [30]. As such, ‘dominant’ community members may be more likely to coevolve, providing any resistance does not diminish competitive ability (which would select against resistant phenotypes) [9,27, 31]. The speed and ease with which bacteria evolve resistance is also likely to determine the relative ability of phages to reciprocally evolve and coexist with their hosts. Phages are typically assumed to be ‘behind’ in coevolution and, therefore, more vulnerable to extinction once resistance evolves [1,9].

Here, we investigated how community context influences ecological and coevolutionary dynamics using a stable three-species bacterial community. This community consists of *Ochrobactrum teleogrylli* AB1, *P. fluorescens* AB1 and *Variovorax* sp. AB1, each of which has a species-specific lytic bacteriophage [32]. Previous work characterizing the community demonstrated that *Ochrobactrum* and *Pseudomonas* have growth rate costs when grown with the other species, while *Variovorax* experiences a growth rate benefit from the other two species. *Ochrobactrum* and *Variovorax* typically ‘dominate’ this community while *Pseudomonas* coexists at lower densities [32]. Consequently, we predict bacteria–phage (co)evolution to be more inhibited for *Pseudomonas* than the other two species. We evolved these species for 8 weeks in the presence and absence of other community members, with and without phage. Using resistance and time-shift assays (measuring bacteria–phage interactions both within and between time points), we examine how the presence of other bacterial species affected bacteria and phage (co)evolutionary interactions.

## Methods

### Experimental evolution

Species isolates were originally obtained from soil and identified as *Ochrobactrum* sp., *Pseudomonas* sp. and *Variovorax* sp. These species have unique colony morphologies when plated onto King’s medium agar (KB agar) and can stably coexist for several weeks [33,34]. In previous work, species-specific bacteriophages (*Ochrobactrum* phage ORM_20, *Pseudomonas* phage CHF7MC and *Variovorax* phage VAC_51) were identified and characterized for each species [32]. The experimental treatments were monoculture, monoculture with phage (each bacterial species individually with only its species-specific bacteriophage), polyculture (all three bacteria present) and polyculture with phage (all three bacteria with all three phages present). Each treatment was replicated six times. Each species was grown from one colony (isolate) in isolation for 2 days in 6 ml growth media [1/64 tryptic soy broth (TSB), diluted with demineralized H₂O], static, at 28 °C in 25-ml glass microcosms with loosened plastic lids. Using one colony to initiate cultures across multiple replicates is a standard approach across many microbial evolution studies [35,37], as it allows us to examine relative treatment differences between cultures with otherwise the same starting conditions. If our study aimed to examine genetic changes or convergent or parallel evolution, then starting evolution lines with separate picked clones may be more appropriate. However, we were simply interested in the treatment effects, and even if this was our aim, our recently published work focussing on the *Variovorax* sp. populations which show a lack of convergent evolution in phage resistance evolution, even when starting with a single colony [38].

Species densities were normalized to ~10^5^ c.f.u. µl^−1^ as described previously [34]. Ten microlitres of diluted culture (~10^6^ c.f.u.) of each bacterial species were added to fresh vials. To phage-present cultures, ~10^4^ p.f.u. were added (MOI 0.01) (starting density 6,667 p.f.u. ml^−1^). Serial 100-fold dilutions (60 µl culture into 6 ml growth media) took place every week for a total of 8 weeks. Culture samples were cryogenically frozen at −70 °C in glycerol (final concentration: 25%) every transfer. Cultures were plated every second transfer onto KB agar and incubated for 2 days at 28 °C to calculate bacteria densities based on their unique morphologies. Additionally, phage extractions were performed every second transfer: 900 µl of culture was vortexed with 100 µl of chloroform. Vials were then centrifuged at 14,000 r.p.m. (21,100 ***g***) for 5 min and the supernatant isolated. Phage densities were calculated via spot assays: phage cultures were diluted, and 10 µl of each dilution was spotted onto soft agar overlays of each ancestral bacterium, with 100 µl of overnight bacterial culture added to 7 ml of soft KB agar. If phages decreased below detectable densities (100 p.f.u. ml^−1^), extracts were amplified in overnight cultures of ancestral strains of each species and re-spotted to confirm extinction. If phage later proved to be recoverable and were simply below detectable density, mean densities of treatment populations were estimated with these populations being given a population density of 100 (the detection limit). Supernatants from no-phage controls were also spotted to test for phage contamination, of which none was detected across all replicates.

### Measuring phage resistance and bacteria–phage coevolution

We estimated phage resistance to ancestral phage for each species from week two cultures. Twelve colonies were picked from each time point, for each bacterium within each treatment. Colonies were inoculated into 150 µl TSB and grown overnight at 28 °C in 96-well plates. Phage resistance was analysed using spot assays as above with ancestral phage.

As only *Ochrobactrum* and its phage coexisted for 8 weeks across mono- and polyculture treatments, we were only able to characterize coevolution for this pair. Twelve bacterial colonies from each treatment replicate from weeks two, four and six were picked and grown as above. Where phage densities appeared extinct in week six replicates of polyculture lines, phage extracts were amplified overnight alongside ancestral *Ochrobactrum* to recover observable densities for coevolution analyses. Changes in phage resistance and infectivity were tested by spotting phages from different timepoints (ancestral, two, four and six) against bacteria from the same, past or future timepoints. If bacteria and phage are coevolving via arms-race dynamics, we expect bacteria to be more resistant to phage from the past and more susceptible to phage from future timepoints [11,39]. If dynamics follow a fluctuating-selection dynamic, we expect bacteria to have greater resistance to contemporary phage populations [1].

### Cost of resistance assay

Next, we considered if phage resistance was costly to *Ochrobactrum’*s and *Variovorax’s* density in monoculture and polyculture. This was not possible for *Pseudomonas* as phage resistance only emerged in two replicates, therefore, not providing enough isolates from independent replicates. Phage-resistant, phage-susceptible and ancestral *Ochrobactrum* and *Variovorax* isolates were grown in monoculture and polyculture with ancestral strains of the other two species, in the absence of phage (six replicates per treatment). For *Ochrobactrum*, six phage-resistant and six phage-susceptible isolates were isolated from independent replicates (one resistant and one susceptible isolate from each replicate) from week two monoculture with phage replicate lines where both genotypes coexisted. For *Variovorax*, six independent phage-resistant isolates and six independent phage-susceptible isolates were isolated from phage and no phage week two monoculture evolution lines, respectively. Colonies were picked from agar plates and inoculated into 150 µl TSB and grown overnight at 28 °C in 96-well plates. Cultures were tested to confirm phage absence in isolated colonies. Phage-susceptible colonies were isolated from ‘no phage’ monoculture evolution lines since phage resistance was too high in the ‘phage’ treatment for susceptible colonies to be isolated from these. Ancestral, phage-resistant and phage-susceptible isolates were individually grown for 2 days at 28 °C, shaking (180 r.p.m.) in 6 ml 1/64 TSB. Ancestral *Ochrobactrum*, *Pseudomonas* and *Variovorax* isolates were also grown in the same conditions. Isolate densities were normalized to 10^5^ c.f.u. μl^−1^ and 10 µl added to relevant vials. Cultures were grown statically for 1 week at 28 °C and then frozen as previously described and plated from frozen. We calculated density change as ln(*N*_1_/ *N*_0_), where *N*_1_ is the final density and *N*_0_ is the starting density. One week is selected as appropriate for comparing results to experimental evolution where cultures are transferred weekly.

### Statistical analyses

All data were analysed using R (v. 4.2.1) in RStudio [40], and all plots were made using the package ‘ggplot2’ [41]. Model simplification was conducted using likelihood ratio tests, and Tukey’s post hoc multiple comparison tests were done using the R package ‘emmeans’ [42]. In a linear mixed effects model, bacterial density (log10 c.f.u. ml^−1^) is analysed against interacting fixed effects of phage presence, treatment (monoculture/polyculture) and time with a random effect of treatment replicate. Similarly, separate models were run for each phage strain analysing phage density (log10 p.f.u. ml^−1^) against interacting fixed effects of treatment and time, with a random effect of treatment replicate. As we were not interested in comparing densities between species, but rather the effects of phage on individual species between treatments, individual models were run for each species.

Phage resistance was analysed for week two cultures in a linear model with the proportion of phage-resistant isolates (arcsine transformed) analysed against interacting effects of treatment (monoculture/polyculture) and species. We further tested whether there was a relationship between the proportion of phage-resistant isolates (arcsine transformed) and population density at week two using a linear model. To assess whether *Ochrobactrum* had coevolved with phage, the proportion of phage-susceptible isolates was analysed in a generalized linear mixed effects model with interacting fixed effects of phage time, bacteria time and treatment (monoculture or polyculture) with a binomial error structure and a random effect of treatment replicate.

The effect of phage resistance on *Ochrobactrum* and *Variovorax* density change (*m*) was estimated using separate linear mixed effects models for each species. Density change (*m*) was analysed against interacting fixed effects of test environment (isolates grown in monoculture and polyculture) and phage resistance (ancestral, resistant and susceptible). For *Ochrobactrum*, a random effect of clonal identity was included (same isolates used across different monoculture and polyculture treatments) nested within the treatment replicate isolates were taken from the evolution experiment. For *Ochrobactrum*, resistant and susceptible clones were isolated from monoculture lines treated with phage – as such, resistant and susceptible isolates coexisted within replicates and were non-independent. For *Variovorax*, a random effect of clonal identity was included. Phage-susceptible clones originated from monocultures evolved without phage, whereas phage-resistant clones were isolated from monocultures evolved with phage. It was not possible to isolate susceptible clones from the same phage-treatment evolution line as resistance reached fixation in most replicates.

## Results

### The effects of phage are species-specific

**Fig. 1.**
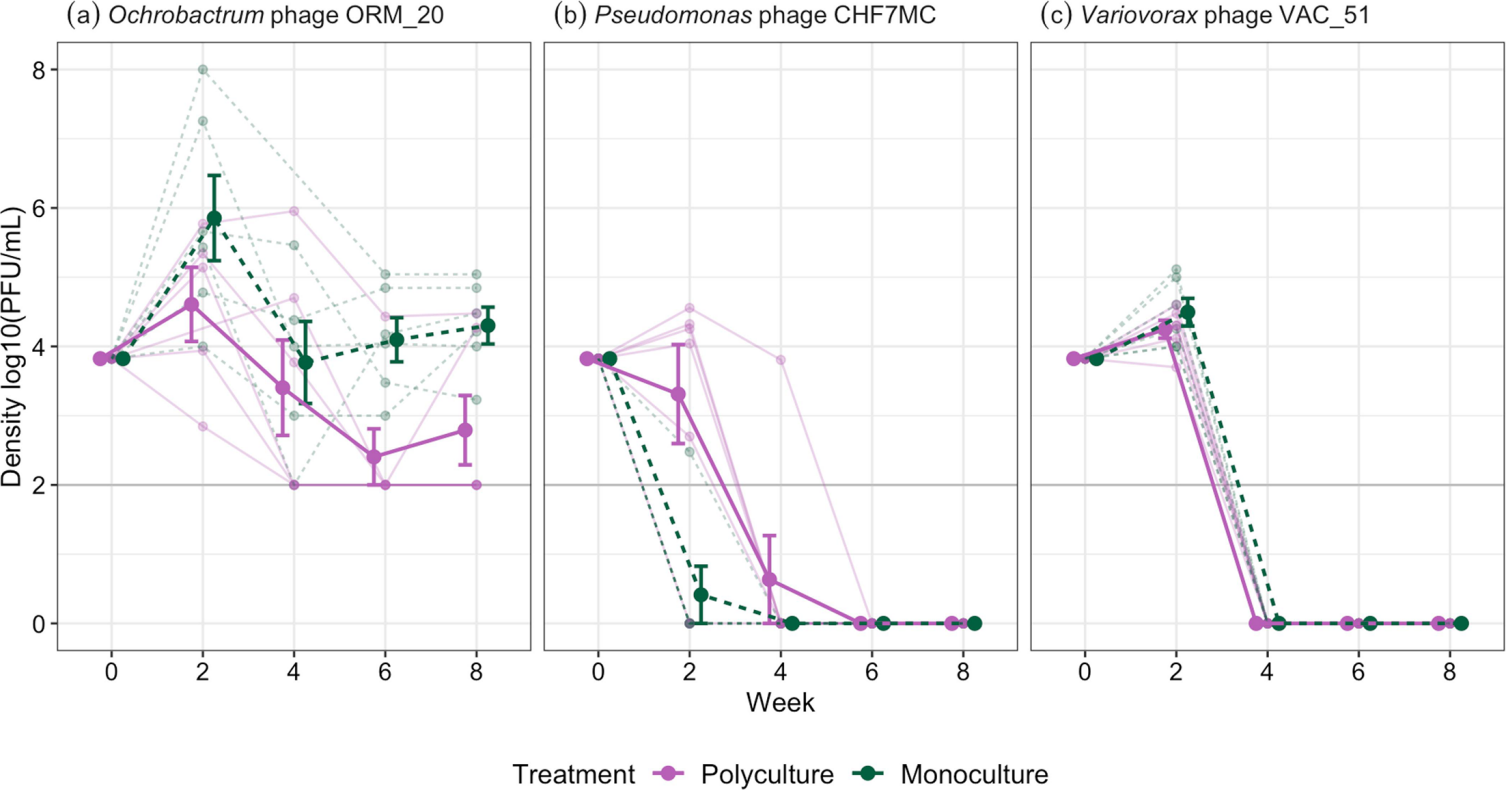
Phage density through time for (**a**) *Ochrobactrum* phage ORM_20, (**b**) *Pseudomonas* phage CHF7MC and (**c**) *Variovorax* phage VAC_51. Limit of detection=100 p.f.u. ml^−1^, which is represented by the horizontal line. Points on this line for ORM_20 indicate phages below detectable density, but phages were not extinct. CHF7MC and VAC_51 were, however, extinct and are therefore plotted at 0. Points with bars represent the means with standard errors. Small points represent separate treatment replicates. Lines connect points from the same treatment replicate.

**Fig. 2.**
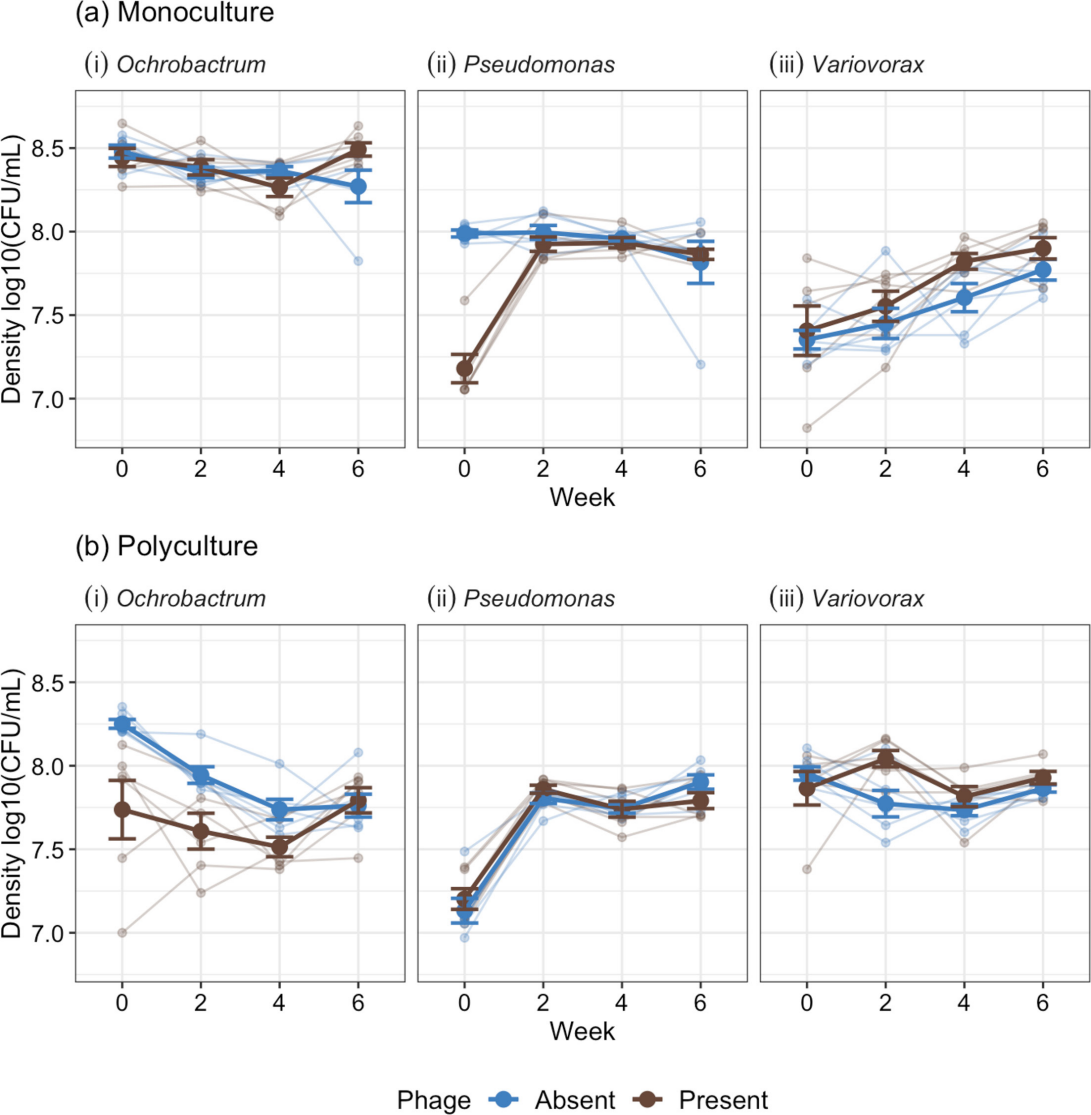
Changes in bacterial density through time with phages present or absent at the start of each experiment in (**a**) monoculture and (**b**) polyculture. Starting bacteria densities=5.22 log10 c.f.u. ml^−1^. Points with bars represent the means with standard errors. Small points represent separate treatment replicates. Lines connect points from the same treatment replicates.

With a three-species synthetic community, we sought to examine multiple bacteria–phage dynamics simultaneously in polyculture and monoculture. *Ochrobactrum* phage ORM_20 density was significantly greater in monoculture ($\bar{x}$= 10^4.48^, 95% CI=10^3.23^–10^5.73^) compared to polyculture ($\bar{x}$= 10^3.3^, 95% CI=10^2.43^–10^4.17^; ANOVA comparing models with and without treatment: $x_{1}^{2}$ = 5.104, *P*=0.024; Tukey HSD (honestly significant difference) comparing densities in polyculture vs. monoculture: estimate=−1.27, t-ratio=−2.29, *P*=0.045), with phages going below detectable densities in 5/6 polyculture replicates at week 6 and 4/6 replicates at week 8; comparatively, phages were always detectable in monoculture lines (Fig. 1a). On average, phage densities declined from week two and then became non-significantly different among weeks four to eight [ANOVA comparing models with and without time: $x_{3}^{2}$ = 21.39, *P*<0.001, Table S1 (available in the online Supplementary Material), Fig. 1a], with this pattern being independent of monoculture or polyculture treatment ($x_{3}^{2}$ = 3.09, *P*=0.378). Lower phage densities in polyculture may be attributable to *Ochrobactrum* densities being reduced under interspecific competition (Tukey HSD comparing *Ochrobactrum* density polyculture and monoculture (no phage) at week two: estimate=−0.444, t-ratio=−7.69, *P*<0.001, Fig. 2b). Phages also significantly lowered bacterial densities in polyculture (phage present: $\bar{x}$= 10^7.66^, 95 % CI=10^7.58^–10^7.75^; phage absent: $\bar{x}$= 10^7.92^, 95% CI=10^7.84^–10^8.01^; ANOVA comparing models with and without treatment and phage interaction: $x_{1}^{2}$ = 11.73, *P*<0.001) while having no significant effect in monoculture (phage present: $\bar{x}$= 10^8.4^, 95% CI=10^8.31^–10^8.48^; phage absent: $\bar{x}$= 10^8.37^, 95 % CI=10^8.28^–10^8.45^; Tukey HSD: *P*>0.05 for weeks two–six, Table S2, Fig. 2a).

For *Pseudomonas* and *Variovorax*, phages went extinct after 2–4 weeks (Fig. 1b and c). Extinction of *Pseudomonas* phage CHF7MC was faster in monoculture compared to polyculture, with only one replicate containing phage in monoculture at week two compared to 5/6 replicates containing phage in polyculture (Fig. 1b). The rapid extinction of *Pseudomonas* phage resulted in short-term effects of phage on bacterial densities in monoculture (phage present: $\bar{x}$= 10^7.18^, 95 % CI=10^7.07^–10^7.29^; phage absent: $\bar{x}$= 10^7.99^, 95 % CI=10^7.88^–10^8.1^) with bacteria densities recovering after week two (ANOVA comparing models with and without a three-way interaction between treatment, time and phage presence: $x_{3}^{2}$ = 55.89, *P*<0.001, Fig. 2b, Table S3). In polyculture, phages did not significantly impact *Pseudomonas* density (phage present: $\bar{x}$= 10^7.2^, 95 % CI=10^7.09^–10^7.31^; phage absent: $\bar{x}$= 10^7.13^, 95% CI=10^7.02^–10^7.24^; Tukey HSD comparisons at week two between phage present/absent cultures: estimate=−0.07, t-ratio=−0.91, *P*-value=0.801); however, this may be due to interspecific competition providing bottom-up control of density (Tukey HSD comparisons at week two between phage present monocultures to phage absent polycultures: estimate=−0.05, t-ratio=−0.62, *P*=0.926), indicating that effects of competition and phage were not additive.

*Variovorax* phage VAC_51 densities were not significantly affected by whether *Variovorax* was in mono- vs. polyculture (ANOVA comparing models with and without treatment × time interaction: $x_{3}^{2}$ = 3.704, *P*=0.295; independent effect of treatment: $x_{1}^{2}$ = 1.17, *P*=0.279), and we detected no phage in any cultures at week four onwards (ANOVA comparing models with and without time: $x_{3}^{2}$ = 217.3, *P*<0.001, Fig. 1c). However, phages had a consistent positive effect on *Variovorax* densities, with cultures that had been exposed to phage reaching significantly higher densities than no-phage cultures (phage present: $\bar{x}$= 10^7.79^ c.f.u. ml^−1^, 95%CI=10^7.74^–10^7.85^; phage absent: $\bar{x}$= 10^7.69^ c.f.u. ml^−1^, 95 % CI=10^7.63^–10^7.74^; ANOVA comparing models with and without phage: $x_{1}^{2}$ = 7.21, *P*=0.007; Tukey HSD between no-phage and phage cultures: estimate=−0.103, t-ratio=−2.71, *P*-value=0.013, Fig. 2c). This effect was evident after phage extinction in both monoculture and polyculture (Fig. 2c, Table S4).

Combined, we have observed species-specific effects of phage that also differed between monocultures and polycultures for two bacterial species. Interactions between bacteria and phages were also asymmetric, with bacteria persisting while 2/3 phages went extinct. For *Pseudomonas*, densities were only affected by phage in monoculture, and effects were absent once phages went extinct, which was also faster in monoculture. *Ochrobactrum* was the only species to coexist with its phage, with bacteria and phage densities significantly lower in polyculture (albeit not extinct), while bacteria densities were unaffected by phage in monoculture. Comparatively, phage had a positive impact on *Variovorax* abundance in monoculture and polyculture, including after phage extinction. There were no differences between monoculture and polyculture regarding *Variovorax* phage density or extinction rates (Fig. 1). The change in bacteria density from their inoculum density to week two is presented in Fig. S1.

### Phage resistance evolution is inhibited in polyculture

**Fig. 3.**
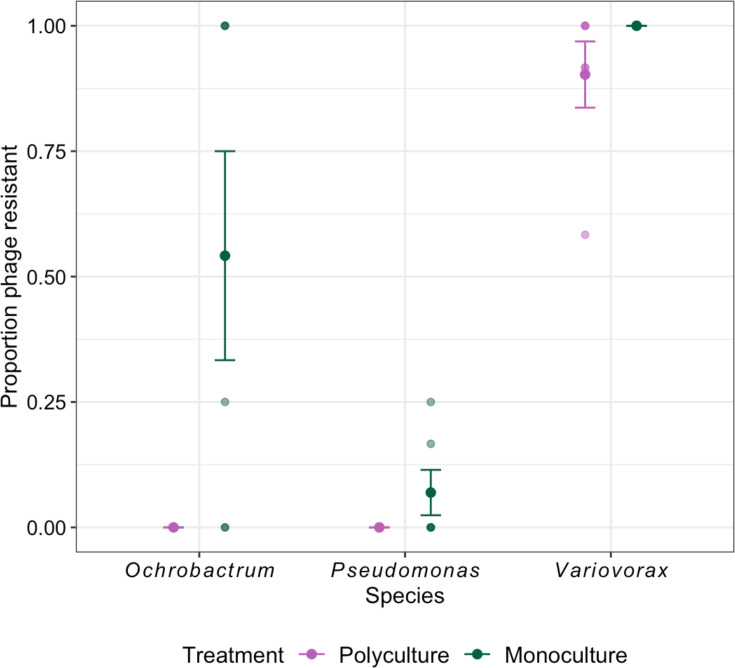
The proportion of phage-resistant bacteria after 2 weeks evolving with phage in polyculture and monoculture. Limit of detection=0.08. Points with bars represent the means with standard errors. Small points represent separate treatment replicates.

We next examined to what extent phage resistance was impacted by community context by comparing bacteria–phage evolution in monoculture and polyculture through time. As *Pseudomonas* and *Variovorax* phages went extinct after 2–4 weeks, we estimated phage resistance for all species at week two. Phage resistance levels were species specific (ANOVA comparing models with and without species × treatment interaction: *F*_2,29_ = 3.40, *P*=0.047, Fig. 3). For *Ochrobactrum*, phage resistance was inhibited in polyculture with no resistant isolates, while in monoculture, 54.2% (SE (standard error) ±20.8) isolates evolved resistance (Tukey HSD comparing resistance in polyculture to monoculture: estimate=−0.873, t-ratio=−3.983, *P*-value<0.001, Fig. 3). Similarly, we found no phage resistance in *Pseudomonas* in polyculture, while 6.94 % (SE±4.52) of isolates were resistant in monoculture, although this was not significantly different (Tukey HSD: estimate=−0.157, t-ratio=−0.753, *P*-value=0.457, Fig. 3). *Variovorax* evolved 100% resistance in monoculture and 90.3% (SE±6.6) resistance in polyculture which was non-significantly different (Tukey HSD: estimate=−0.215, t-ratio=−1.027, *P*-value=0.313, Fig. 3). These results show inhibited evolution of resistance across all species in polyculture, but this effect was largest for *Ochrobactrum*. Furthermore, this seemed unrelated to the ‘relative abundance’ of each species within a community with no resistance evolution for *Ochrobactrum* and high levels of resistance for *Variovorax* (the dominant community members [34]) and overall low resistance evolution for *Pseudomonas* in both polyculture and monoculture. Indeed, there is no significant relationship between the proportion of resistance in the population and population density (*F*_1,33_ = 2.82, *P*=0.103).

### Lack of resistance evolution in polyculture is not associated with resistance costs

**Fig. 4.**
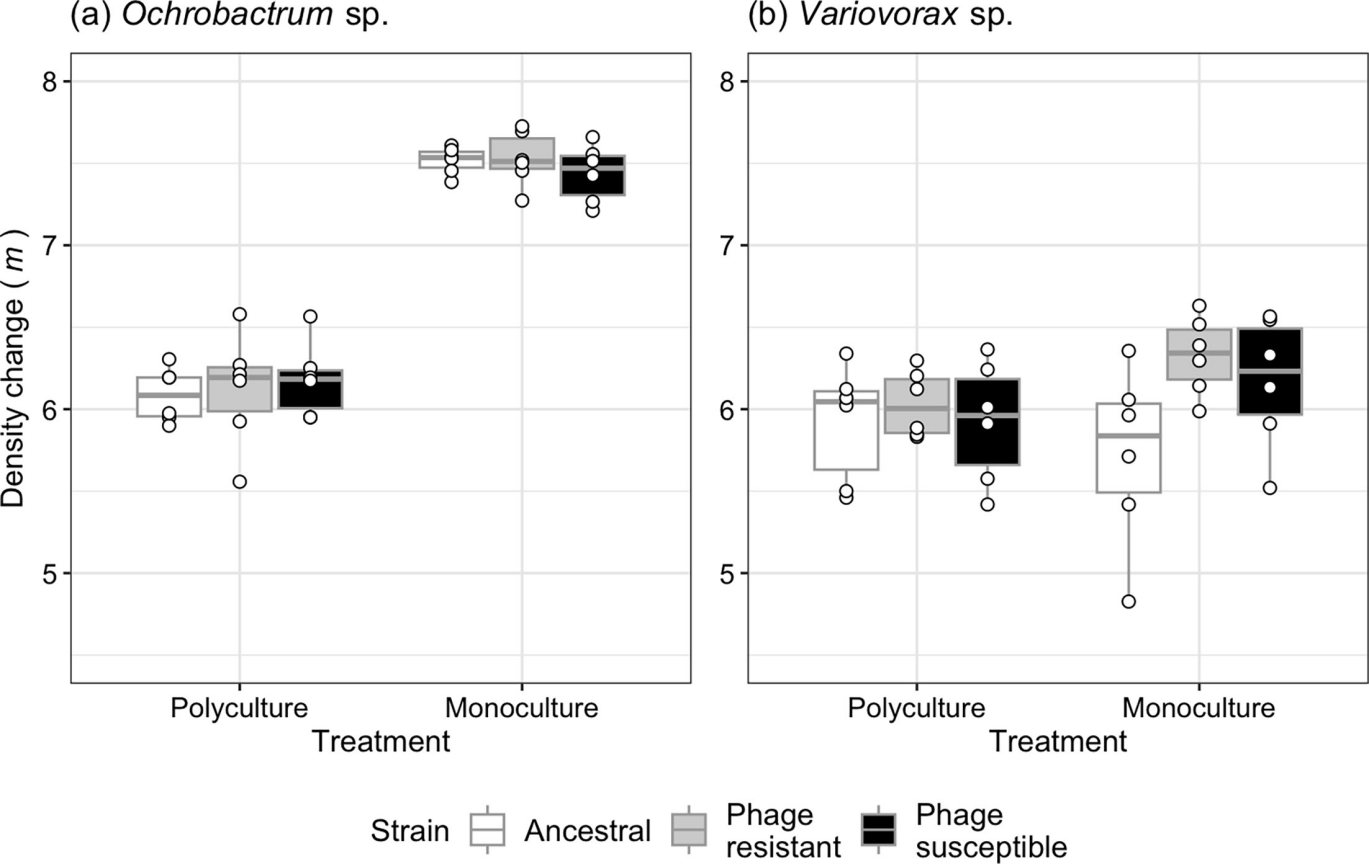
Density change after 1 week of different (**a**) *Ochrobactrum* and (**b**) *Variovorax* strains, including ancestral, phage-resistant and phage-susceptible isolates, grown in polyculture and monoculture for 1 week. Points represent individual treatment replicates. Tops and bottoms of the bars represent the 75th and 25th percentiles of the data, white lines indicate the medians, and whiskers extend from their respective hinge to the smallest or largest value no further than 1.5×the interquartile range.

It is not unusual for interspecific interactions to decrease the evolution of phage resistance, with mechanisms ranging from reduced bacteria-phage contact rates, lower mutation supply rates or selection against less-fit resistant mutants [9]. In *Ochrobactrum* especially, we found that bacterial densities were significantly lower in polyculture when phages were added. With this result coinciding with lower rates of phage resistance, this could indicate context-specific resistance costs or density effects reducing mutation supply rates and/or encounter rates [16,20, 43]. We examined whether interspecific competitors had competitively excluded phage-resistant *Ochrobactrum* and *Variovorax* isolates owing to resistance costs to density change. This was not possible for *Pseudomonas* due to the low rates of resistance evolution. To this end, phage-susceptible and resistant isolates were grown in monoculture and polyculture in the absence of phage, and their densities were compared after 1 week. Density change after 1 week was the selected fitness measure as this reflects the transfer regime of the experiment – mutants of high density at this time point would be transferred onto the following week and, therefore, have a fitness advantage (regardless of changes to other measures of fitness). An example of how high densities at the 1 week stage lead to a fitness advantage is evidenced by our work with *Variovorax* sp., where phage-resistant isolates had a higher density in the death phase at 7 days, albeit without changes to exponential growth measures of fitness [38]. Here, the relative density change of *Ochrobactrum* was non-significantly different between ancestral, phage-resistant and susceptible isolates (ANOVA comparing models with and without treatment (monoculture, polyculture) × phage resistance: $\chi_{2}^{2}$ = 1.48, *P*=0.476; phage resistance: $\chi_{2}^{2}$ = 0.081, *P*=0.994, Fig. 4a) and *Variovorax* (ANOVA comparing models with and without treatment (monoculture, polyculture) × phage resistance: $\chi_{2}^{2}$ = 5.71, *P*=0.057; phage resistance: $\chi_{2}^{2}$ = 3.4, *P*=0.183, Fig. 4b). Both species density changes were significantly lower in polyculture compared to monoculture (*Ochrobactrum*: ANOVA comparing models with and without treatment: $\chi_{2}^{2}$ = 93.04, *P*<0.001; Tukey HSD comparing density change in polyculture vs monoculture: estimate=−1.36, t-ratio=−20.23, *P*<0.001. *Variovorax*: ANOVA: $\chi_{2}^{2}$ = 25.84, *P*<0.001; Tukey HSD: estimate=−0.267, t-ratio=−10.63, *P*<0.001, Fig. 4). Consequently, lower rates of phage resistance in polyculture were not due to resistance costs in interspecific competition and are more likely explained by lower mutation supply rates and/or lower encounter rates.

### Coevolutionary dynamics of *Ochrobactrum* and phage

**Fig. 5.**
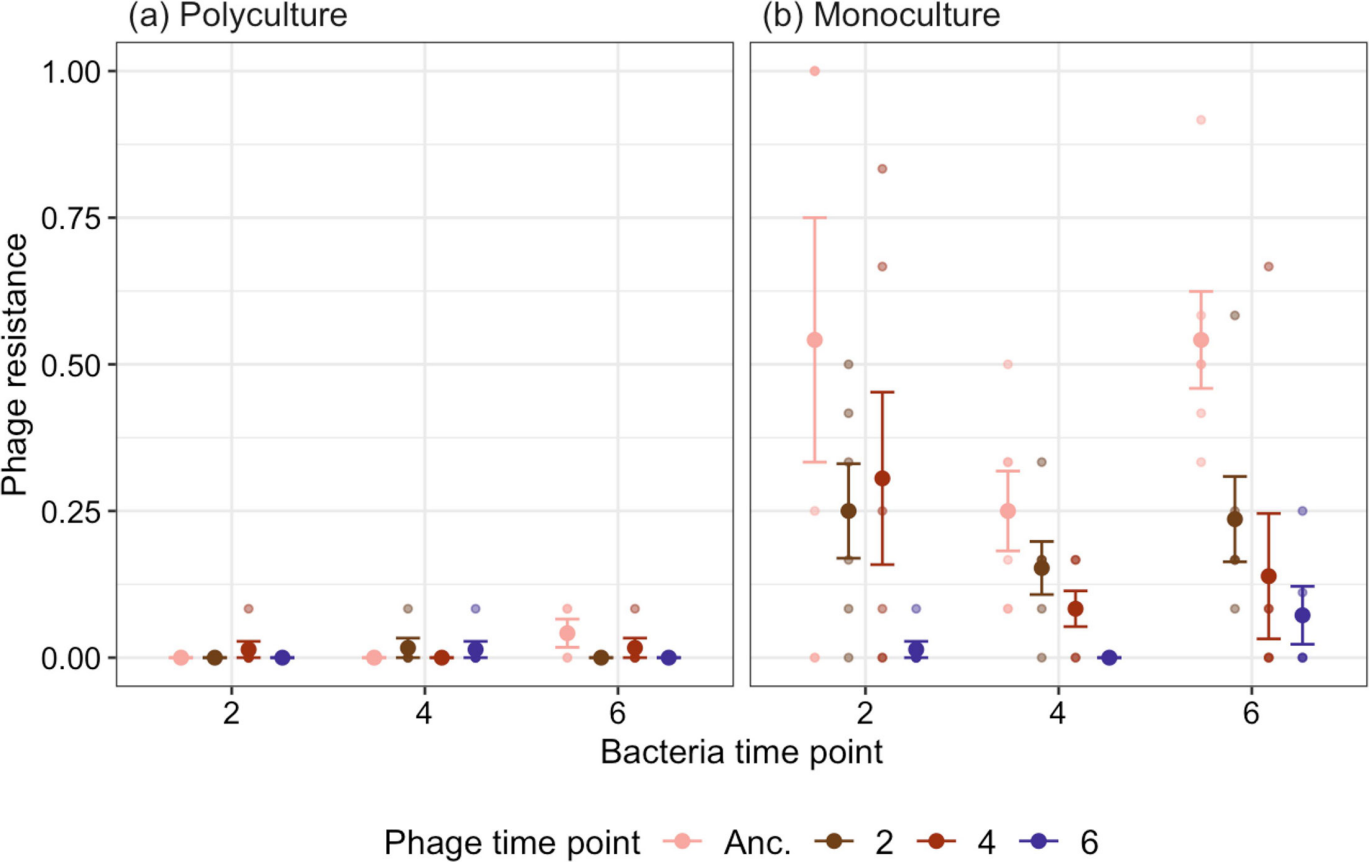
Changes in phage resistance (the proportion of phage-resistant bacteria) in *Ochrobactrum* through time (weeks), demonstrated by assaying phages from different time-points against bacteria from different time-points. Twelve bacterial isolates were assayed for resistance within each treatment replicate [6]. Points with bars represent the means with standard errors. Small points represent separate treatment replicates.

As only *Ochrobactrum* coexisted with its phage (Figs1a  2a), we determined if coevolution had occurred by measuring the resistance of bacteria to phage both within and between time points. In polyculture, low-frequency resistance (4.17%, SE±2.4 %) was detected against ancestral phage only at week six, ruling out significant coevolution. In monoculture, 54.2% (SE±20.8 %) of isolates were resistant to the ancestral phage by week two. Phage infectivity increased (resistance decreased to evolved phage) through time (ANOVA comparing models with and without phage time: $\chi_{3}^{2}$ = 122.7, *P*<0.001; Tukey HSD comparing phage infectivity between ancestral phage, time-points two or four to six: *P*<0.001, Fig. 5). Mean bacterial resistance declined at week four (ANOVA comparing models with and without bacterial time-point: $\chi_{2}^{2}$ = 27.52, *P*<0.001; Tukey HSD comparing resistance between weeks two and four: estimate=1.12, z-ratio=4.76, *P*<0.001; weeks four and six: estimate=−0.972, z-ratio=−4.09, *P*<0.001) and then increased at week six back to levels non-significantly different to week two (Tukey HSD weeks two and six: estimate=0.15, z-ratio=0.72, *P*=0.752). However, bacteria from each time point showed similar patterns of resistance to phages isolated from past, contemporary and future time-points, being more resistant to ancestral phage and more susceptible to phage isolated from later time-points (ANOVA comparing models with and without phage time × bacteria time interaction: $\chi_{6}^{2}$ = 11.36, *P*=0.078, Fig. 5); for example, bacteria from time-point two had resistance levels to phage from time-point two as bacteria from time-point four. These results suggest limited asymmetrical coevolution in monoculture with bacteria evolving resistance to phage (although resistance did not go to fixation) and phage slowly evolving increased infectivity against a subset of bacterial mutants through time.

## Discussion

Over 8 weeks of experimental evolution, we examined ecological and (co)evolutionary dynamics between three bacteria and phage pairs in monoculture (single pairs) and polyculture (all pairs coexisting). Overall, interactions between bacteria and phages were species-specific in effects on density, resistance evolution and (co)evolution. *Ochrobactrum* showed the biggest difference between monoculture and polyculture, with densities only affected by phage in polyculture, while coevolution only occurred in monoculture. Across all species, resistance evolution was inhibited in polyculture (Fig. 3), although this effect was only significant for *Ochrobactrum*. This suggests that the probability of (co)evolution is highly dependent on host–phage pair, with community context being inhibitory of resistance evolution and consequently coevolution. While previous studies have examined bacteria and phage (co)evolution in polyculture [9], ours is unique in examining (co)evolutionary and ecological dynamics across all stably coexisting pairs, as is the case in nature. Consequently, phage resistance and coevolution may be rarer than what is predicted based on the use of model organisms.

Ecological dynamics between bacteria and phage in polyculture and monoculture were species-specific. While *Pseudomonas* densities were initially impacted by phage, densities recovered following phage extinction at week two. *Pseudomonas* phage CHF7MC is closely related to strain CHF7 which infects *P. syringae*, suggesting this phage may not be well-adapted to this *Pseudomonas* strain as extinction occurred without resistance [32]. *Variovorax* densities significantly increased in the presence of phage, with this effect evident with fixation of phage resistance, followed by phage extinction at week two. Phages have been shown to increase densities in other systems [14], but exploration of this mechanism is beyond the scope of this project [44]. In contrast, *Ochrobactrum*’s densities were significantly reduced in polyculture when phages were present, and this was the only bacteria–phage pair to coexist for the study duration. This density decline is consistent with an interaction between phage lysis and interspecific competition as densities were unaffected in monoculture. Competitive interactions have been shown to increase the effects of phage on bacteria density [45]. Although *Ochrobactrum* evolved phage resistance in monoculture, which may have buffered densities against phage [46], its phage also increased in infectivity through time (92.8 % week six isolates susceptible to contemporary phage) without affecting *Ochrobactrum* densities. As *Ochrobactrum* did not reciprocally increase in resistance, and densities did not decline, this would suggest that resistance alone did not buffer *Ochrobactrum* against the effects of its phage.

Across all species, resistance evolution was inhibited in polyculture (although only significantly different for *Ochrobactrum*, Fig. 3), despite resistance evolution in monoculture being very different between species. Consistent with previous research, phage resistance rates were lower in polyculture compared to monoculture [29]. This effect was most evident for *Ochrobactrum* which coevolved with its phage in monoculture while not starting to evolve phage resistance until week six in polyculture (4.17%, SE±2.4%). *Variovorax*’*s* and *Ochrobactrum*’*s* lowered resistance in polyculture was not attributed to phage resistance costs to interspecific competition (in either polyculture or monoculture) and was most likely due to lowered bacteria–phage contact rates or mutation-supply rates [17,35, 47, 48]. Although we were unable to examine the mechanism of lowered resistance evolution in polyculture for *Pseudomonas*, bottom-up control of *Pseudomonas*’s density would have reduced contact and mutation rates in a similar manner to the other two species.

Bacteria–phage coevolution was evident for one pair, *Ochrobactrum* and its phage, in monoculture but not polyculture. While it is commonly assumed that bacteria and phage coevolve, this is perhaps less likely in complex systems if resistance is unlikely to evolve ([9], although see [14,24]). As studies typically focus on bacteria–phage pairs that are known to coevolve, it is less understood how common coevolution is in wider systems. Particularly in this case, where phages are poorly infectious (*Pseudomonas*) or resistance can evolve rapidly and reach fixation (*Variovorax*), it is less likely that coevolution will occur.

That results contrast in monoculture and polyculture has important implications. Even in monoculture, each bacteria–phage interaction was species-specific, and coevolution only occurred in one bacteria–phage pair. This emphasizes the difficulty in generalizing from model systems and in focusing on model systems that behave predictably (e.g. always coevolve). Across all pairs, resistance evolution was inhibited in polyculture, which further emphasizes the difficulty in generalizing results from less-natural monoculture conditions to complex communities. This is particularly important in the context of phage therapy, where phages are screened in monoculture for ones which reduce bacteria densities the most and result in the lowest rates of resistance [49]. Although contact rates between bacteria and phage are typically high in phage therapy (therefore increasing selection), resistance may still be less likely *in vivo* owing to reduced bacteria replication rates and context-dependent resistance costs [31]. High rates of phage extinction for two out of three phages also imply that bacteria and phage ecological dynamics may be more unstable in the long term than what is assumed by theory explaining how phages mediate bacteria densities in communities. Here, phages are assumed to regulate bacteria densities and increase diversity, while our research instead suggests bacteria can outgrow their phage counterparts [9,50]. The instability of the phages in culture would appear to depend on how adapted the phage is to the bacterial host (as likely to be the case for *Pseudomonas*) and the ability of phage-resistant mutants to dominate populations (as with *Variovorax*). Polyculture conditions may increase the likelihood of extinction if contact rates are reduced (as in the case with *Ochrobactrum* phage decreasing below detectable density although not extinct), with literature suggesting low contact rates between hosts and phage in nature [51]. Although our community is still simplified compared to natural communities, by examining how multiple species evolve and interact simultaneously with their phages, our results have more ecological relevance than previous research focussing on single pairs.

## Supplementary material

10.1099/mic.0.001577 Supplementary Material 1.

## Abbreviations

CFU: colony forming unit
HSD: honestly significant difference
KB: King's medium
MOI: multiplicity of infection
PFU: plaque forming unit
TSB: tryptic soy broth
