# Corrigendum: Bacteria–phage (co)evolution is constrained in a synthetic community across multiple bacteria–phage pairs

**DOI:** 10.1099/mic.0.001645

**Published:** 2025-12-04

**Authors:** Meaghan Castledine, Daniel Padfield, Marli Schoeman, Amy Berry, Angus Buckling

**Affiliations:** 1 College of Life and Environmental Sciences, Environment and Sustainability Institute, University of Exeter, Penryn, Cornwall, TR10 9EZ, UK; 2 Falmouth School, Trescobeas Rd, Falmouth TR11 4LH, UK

In the published version of the article, the scale of x-axes of Figure 2 are incorrect. The x-axes ranged from 0–6.

The correct x-axes scale should have ranged from 2–8, as shown below:

**Fig. 2**. Changes in bacterial density through time with phages present or absent at the start of each experiment in (**a**) monoculture and (**b**) polyculture. Starting bacteria densities=5.22 log10 c.f.u. ml^−1^. Points with bars represent the means with standard errors. Small points represent separate treatment replicates. Lines connect points from the same treatment replicates.

The authors apologise for any inconvenience caused.

