# Supplementary material for: Bacteria–phage (co)evolution is constrained in a synthetic community across multiple bacteria–phage pairs
Source: Microbiology (Reading). 2025 Jun 19;171(6):001577. doi: 10.1099/mic.0.001577 (PMC12178565; doi:10.1099/mic.0.001577)
Supplement: Supplementary Material 1. [file mic-171-01577-s001.pdf]

Table S1. Densities of *Ochrobactrum* phage ORM_20 through time averaged over polyculture and monoculture. P-values are adjusted by the tukey method for comparing a family of four estimates.

Contrast	Estimate	t-ratio	p-value
2 - 4	1.512	3.971	0.002
2 - 6	1.981	5.352	<0.001
2 - 8	1.686	4.554	<0.001
4 - 6	0.469	1.266	0.591
4 - 8	0.173	0.468	0.965
6 - 8	-0.295	-0.821	0.844

Table S2. Tukey HSD contrasts comparing *Ochrobactrum* densities across polyculture (‘Poly.”) and monoculture (“Mono.”) in the presence and absence of phage. P-values are adjusted by the tukey method for comparing a family of four estimates.

Contrast	Week	Estimate	t-ratio	p-value
Phage absent Poly. - Phage present Poly.	2	0.419	4.949	<0.001
Phage absent Poly. - Phage absent Mono.	2	-0.444	-7.691	<0.001
Phage absent Poly. - Phage present Mono.	2	-0.314	-3.712	0.002
Phage present Poly. - Phage absent Mono.	2	-0.863	-10.188	<0.001
Phage present Poly. - Phage present Mono.	2	-0.733	-12.713	<0.001
Phage absent Mono. - Phage present Mono.	2	0.129	1.528	0.427
Phage absent Poly. - Phage present Poly.	4	0.298	3.516	0.004
Phage absent Poly. - Phage absent Mono.	4	-0.444	-7.691	<0.001
Phage absent Poly. - Phage present Mono.	4	-0.436	-5.145	<0.001
Phage present Poly. - Phage absent Mono.	4	-0.741	-8.755	<0.001
Phage present Poly. - Phage present Mono.	4	-0.733	-12.713	<0.001
Phage absent Mono. - Phage present Mono.	4	0.008	0.095	1
Phage absent Poly. - Phage present Poly.	6	0.306	3.616	0.003
Phage absent Poly. - Phage absent Mono.	6	-0.444	-7.691	<0.001
Phage absent Poly. - Phage present Mono.	6	-0.427	-5.045	<0.001
Phage present Poly. - Phage absent Mono.	6	-0.750	-8.855	<0.001
Phage present Poly. - Phage present Mono.	6	-0.733	-12.713	<0.001
Phage absent Mono. - Phage present Mono.	6	0.016	0.195	0.997
Phage absent Poly. - Phage present Poly.	8	0.018	0.217	0.996
Phage absent Poly. - Phage absent Mono.	8	-0.444	-7.691	<0.001
Phage absent Poly. - Phage present Mono.	8	-0.715	-8.443	<0.001
Phage present Poly. - Phage absent Mono.	8	-0.462	-5.456	<0.001
Phage present Poly. - Phage present Mono.	8	-0.733	-12.713	<0.001
Phage absent Mono. - Phage present Mono.	8	-0.271	-3.204	0.011

Table S3. Tukey HSD contrasts comparing *Pseudomonas* densities across polyculture (‘Poly.”) and monoculture (“Mono.”) in the presence and absence of phage. P-values are adjusted by the tukey method for comparing a family of four estimates.

Contrast	Week	Estimate	t-ratio	p-value
Phage absent Poly. - Phage present Poly.	2	-0.070	-0.908	0.801
Phage absent Poly. - Phage absent Mono.	2	-0.856	-11.155	<0.001
Phage absent Poly. - Phage present Mono.	2	-0.048	-0.619	0.926
Phage present Poly. - Phage absent Mono.	2	-0.787	-10.247	<0.001
Phage present Poly. - Phage present Mono.	2	0.022	0.289	0.992
Phage absent Mono. - Phage present Mono.	2	0.809	10.536	<0.001
Phage absent Poly. - Phage present Poly.	4	-0.053	-0.696	0.898
Phage absent Poly. - Phage absent Mono.	4	-0.189	-2.465	0.075
Phage absent Poly. - Phage present Mono.	4	-0.119	-1.549	0.414
Phage present Poly. - Phage absent Mono.	4	-0.136	-1.770	0.297
Phage present Poly. - Phage present Mono.	4	-0.065	-0.853	0.829
Phage absent Mono. - Phage present Mono.	4	0.070	0.916	0.796
Phage absent Poly. - Phage present Poly.	6	0.004	0.053	1
Phage absent Poly. - Phage absent Mono.	6	-0.214	-2.790	0.034
Phage absent Poly. - Phage present Mono.	6	-0.190	-2.470	0.074
Phage present Poly. - Phage absent Mono.	6	-0.218	-2.843	0.029
Phage present Poly. - Phage present Mono.	6	-0.194	-2.523	0.065
Phage absent Mono. - Phage present Mono.	6	0.025	0.320	0.989
Phage absent Poly. - Phage present Poly.	8	0.111	1.451	0.473
Phage absent Poly. - Phage absent Mono.	8	0.087	1.128	0.674
Phage absent Poly. - Phage present Mono.	8	0.039	0.505	0.958
Phage present Poly. - Phage absent Mono.	8	-0.025	-0.323	0.988
Phage present Poly. - Phage present Mono.	8	-0.073	-0.946	0.78
Phage absent Mono. - Phage present Mono.	8	-0.048	-0.623	0.924

Table S4. Tukey HSD contrasts comparing *Variovorax* densities in polyculture (poly.) and monoculture (mono.) through time. P-values adjusted using the Tukey method of comparing a family of three estimates.

Contrast	Week	Estimate	t-ratio	p-value
Poly. - Mono.	2	0.530	7.202	<0.001
Poly. - Mono.	4	0.406	5.519	<0.001
Poly. - Mono.	6	0.062	0.842	0.402
Poly. - Mono.	8	0.060	0.822	0.413

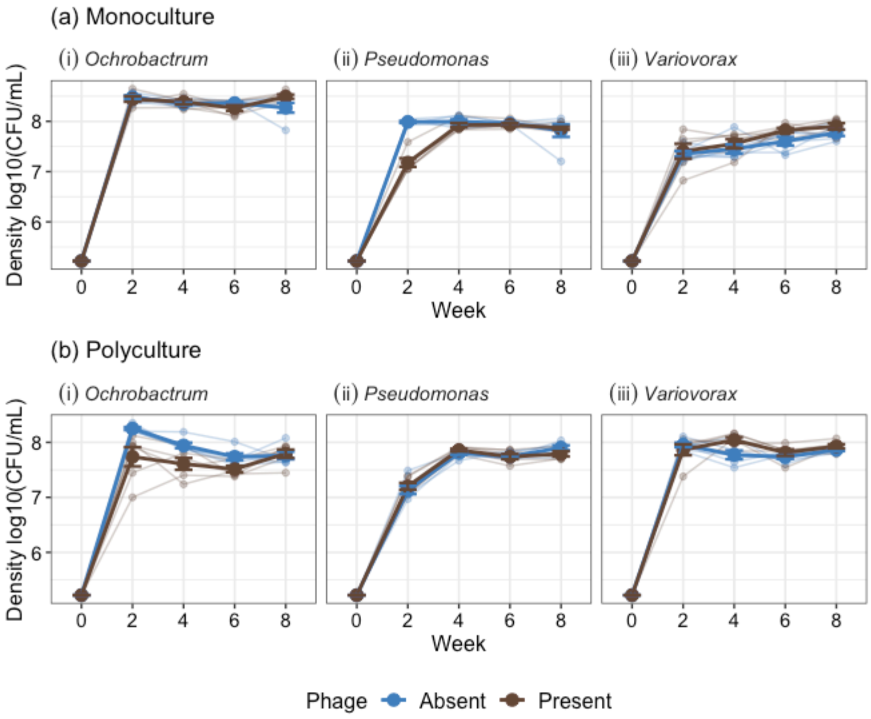

Figure S1. Changes in bacterial density through time with phages present or absent at the start of each experiment in (a) monoculture and (b) polyculture. Week 0 indicates the population density from inoculation of starting bacteria populations to equilibrium density by week 2. Points with bars represent the means with standard errors. Small points represent separate treatment replicates. Lines connect points from the same treatment replicate.
